# The deubiquitinating enzyme USP35 regulates the stability of NRF2 protein

**DOI:** 10.1515/biol-2022-0935

**Published:** 2024-08-16

**Authors:** Dian Zhang, Jiawen Li, Chao Zhang, Jinliang Xue, Peihao Li, Kai Shang, Xiao Zhang, Baoping Lang

**Affiliations:** Department of Thoracic Surgery, Luoyang Central Hospital Affiliated to Zhengzhou University, Xigong District, Luoyang, China

**Keywords:** NRF2, USP35, deubiquitylation, chemoresistance, esophageal cancer

## Abstract

Many cancers exhibit resistance to chemotherapy, resulting in a poor prognosis. The transcription factor NRF2, activated in response to cellular antioxidants, plays a crucial role in cell survival, proliferation, and resistance to chemotherapy. This factor may serve as a promising target for therapeutic interventions in esophageal carcinoma. Recent research suggests that NRF2 activity is modulated by ubiquitination mediated by the KEAP1-CUL3 E3 ligase complex, highlighting the importance of deubiquitination. However, the specific deubiquitinase responsible for regulating NRF2 in esophageal cancer remains unknown. In this study, a novel regulator of the NRF2 protein, Ubiquitin-Specific Protease 35 (USP35), has been identified. Mechanistically, USP35 modulates NRF2 stability through enzymatic deubiquitination. USP35 interacts with NRF2 and facilitates its deubiquitination. Knockdown of USP35 leads to a notable increase in NRF2 levels and enhances the sensitivity of cells to chemotherapy. These findings suggest that the USP35-NRF2 axis is a key player in the regulation of therapeutic strategies for esophageal cancer.

## 1 Introduction

NRF2 is recognized as a key transcription factor that orchestrates the cellular antioxidant response. Recent studies have revealed multiple functions of NRF2 beyond redox regulation. Over the past two decades, research on NRF2 has increasingly focused on its potential as a primary target for cancer prevention and treatment. The expanding understanding of NRF2’s role in cancer therapy has presented novel challenges and opportunities in the field. NRF2, a Cap’n’collar (CNC) leucine zipper (bZIP) transcription factor, is composed of seven Neh domains, each serving a distinct function [1]. Through heterodimerization with small MAF proteins, the NRF2 protein regulates the expression of over 200 genes containing antioxidant response elements (AREs) [2]. NRF2 plays a critical role in controlling a wide array of biochemical processes and metabolic functions, including drug metabolism and excretion, iron metabolism, energetic metabolism, amino acid metabolism, autophagy, proliferation, survival, DNA repair, mitochondrial physiology, and proteasomal degradation [1,3,4].

The ubiquitin-proteasome system (UPS) plays a critical role in the regulation of numerous cellular functions, including cell growth, proliferation, and DNA repair [5,6]. Recent research has highlighted the significant impact of the UPS on various human diseases [7,8]. Post-translational modifications, such as ubiquitination, are involved in the modulation of protein stability and activity, influencing a wide range of biological processes, including tumorigenesis and tumor development [9–11]. Ubiquitination is catalyzed by the concertive actions of E1 activating enzymes, E2 conjugating enzymes, and E3 ligating enzymes, which covalently add ubiquitin to target proteins, leading to diverse biological outcomes, especially proteasomal degradation [12–14]. While NRF2 is ubiquitously expressed in all cell types, its basal protein levels typically remain low in the absence of stress. Three E3 ubiquitin ligases, including KEAP1-CUL3-RBX1 [15–19], β-TrCP-SKP1-CUL1-RBX1 [20,21], and HRD1 [22], have been identified as regulators of NRF2 ubiquitylation and degradation. Despite the extensive research on E3 ubiquitin ligases, the deubiquitinating enzymes (DUBs) responsible for NRF2 regulation have not been thoroughly investigated. Therefore, identifying the DUB(s) of NRF2 is crucial for a more efficient and comprehensive exploration of the NRF2-ARE signaling regulatory network.

A screening of an expression library comprising 56 DUBs was conducted to determine the enzyme responsible for regulating NRF2 stability. Our study revealed that ubiquitin-specific protease 35 (USP35) modulates NRF2 protein stability through its enzymatic activity by interacting with NRF2 and facilitating its deubiquitination. Furthermore, silencing of USP35 resulted in enhanced NRF2 turnover and increased cellular susceptibility to chemotherapy. Based on our findings, USP35 is a new type of DUB that is essential in sensitivity to chemotherapy, and may offer a potential clinical treatment for esophageal cancer.

## 2 Materials and methods

### 2.1 Cell culture

As for the HEK293T and TE-1 cell lines, they were from the Cell Bank of the Chinese Academy of Sciences (Shanghai, China), and the KYSE-410 cell line was from Procell Life Science & Technology Co., Ltd (Hunan, China). A cell line called ECA-109 was purchased from iCell Bioscience. In addition to DMEM (HyClone), the cells were maintained in a solution containing 10% fetal bovine serum, 100 mg/ml streptomycin, 100 IU penicillin, and 4 mM l-glutamine. Cells are cultured at 37°C in 5% CO_2_ humidified incubators.

### 2.2 Reagents

We obtained anti-USP35 (24559-1-AP), anti-NRE2L2 (NRF2) (16396-1-AP), anti-Flag (20543-1-AP), anti-HA (51064-2-AP), rabbit anti-MYC (16286-1-AP), anti-GAPDH (60004-1-Ig), and anti-actin antibodies (66009-1-Ig) from Proteintech. Promega Company provided a CellTiter-Glo Luminescent Cell Viability Assay Kit and Caspa-Glo 3/7 Assay Kit. Selleck Company provided cycloheximide (CHX). MCE provided MG-132 and Cisplatin. Beyotime Company provided TRIzol reagent, TIANGEN Company provided qPCR kit (SYBR Green), Transgene supplied EL transfection reagent, Takara provided exonuclease III used for gene cloning.

### 2.3 Plasmids

Specifically, for lentivirus-based shRNA expression vectors, DNA oligonucleotides containing shRNA sequences were designed and cloned into the pLV-H1-EF1α-puro expression vector. The shRNA sequences targeting USP35 that were used in the study were as follows:

USP35-sh1: GGATAGAGAGGGAGGAAGA;

USP35-sh2; GTGGAGAAGGAGACAGAAA.

### 2.4 CRISPR/Cas9 knockout (KO) cell line

An online tool called CRISPR Design Tool was used to design single-guide RNA (sgRNA) that was then cloned into lentiCRISPRv2 (Addgene, #52961) using the CRISPR Design Tool. The sgRNA sequence for NRF2 was 5ʹ-TAGTTGTAACTGAGCGAAAA-3ʹ.

### 2.5 Transfection and DUBs screening

During the screening of DUBs, every DUB expression plasmid was co-transfected with NRF2 into 293T cells using the EL Transfection Reagent from transgene company. Next, the cells were lysed and examined using antibodies against HA (NRF2), Flag (DUBS), and ACTIN.

### 2.6 RNA extraction and real-time PCR analysis

In the same manner as described previously, RNA was extracted and real-time PCR was carried out [23,24]. Isolate total RNA from cells using TRIzol reagent according to the manufacturer’s instructions. With FastKing gRNA Dispelling RT SuperMix (TIANGEN), cDNA was synthesized from each RNA sample. The levels of RNA expression were normalized to GAPDH, a control gene. FastFire qPCR PreMix SYBR Green (TIANGEN) was used for the real-time PCR.

### 2.7 Ubiquitination assays

NRF2 ubiquitylation was determined by co-transfecting 293T cells with NRF2-HA and Flag-Ub, in the presence or absence of USP35 and its mutants. MG132 (10 M) was added to the cells for 6 h prior to harvesting, followed by RIPA buffer lysis, followed by immunoprecipitation of NRF2 with anti-HA beads. SDS-PAGE was performed on immunoprecipitated NRF2–HA, and anti-Flag antibodies were used to determine NRF2 ubiquitylation.

### 2.8 Immunoprecipitation

Protein interactions can be assessed using immunoprecipitation. After washing with cold PBS and lysing with protease inhibitor rich lysis buffer, cells were centrifuged. Centrifuging the supernatant of the cell lysate at 4°C for 30 min at 11,000*g* removed the supernatant from the cell lysate. As mentioned earlier [23,24], a M2 bead or an anti-HA bead was used in the immunoprecipitation step.

### 2.9 Cell viability assay

To determine the cell viability, approximately 2.5 × 10^4^ cells were seeded into 96-well plates and incubated overnight according to the kit’s instructions. The cells were processed for an appropriate period of time whether they contain or do not contain related compounds. The 96-well plate was then incubated for 30 min at room temperature, followed by addition of equal volumes of CellTiter-Glo reagent and shaking for 2 min. After 15 min of further incubation at room temperature, the luminescence value was recorded.

## 3 Results

### 3.1 USP35 regulates the stability of NRF2 protein

**Figure 1:**
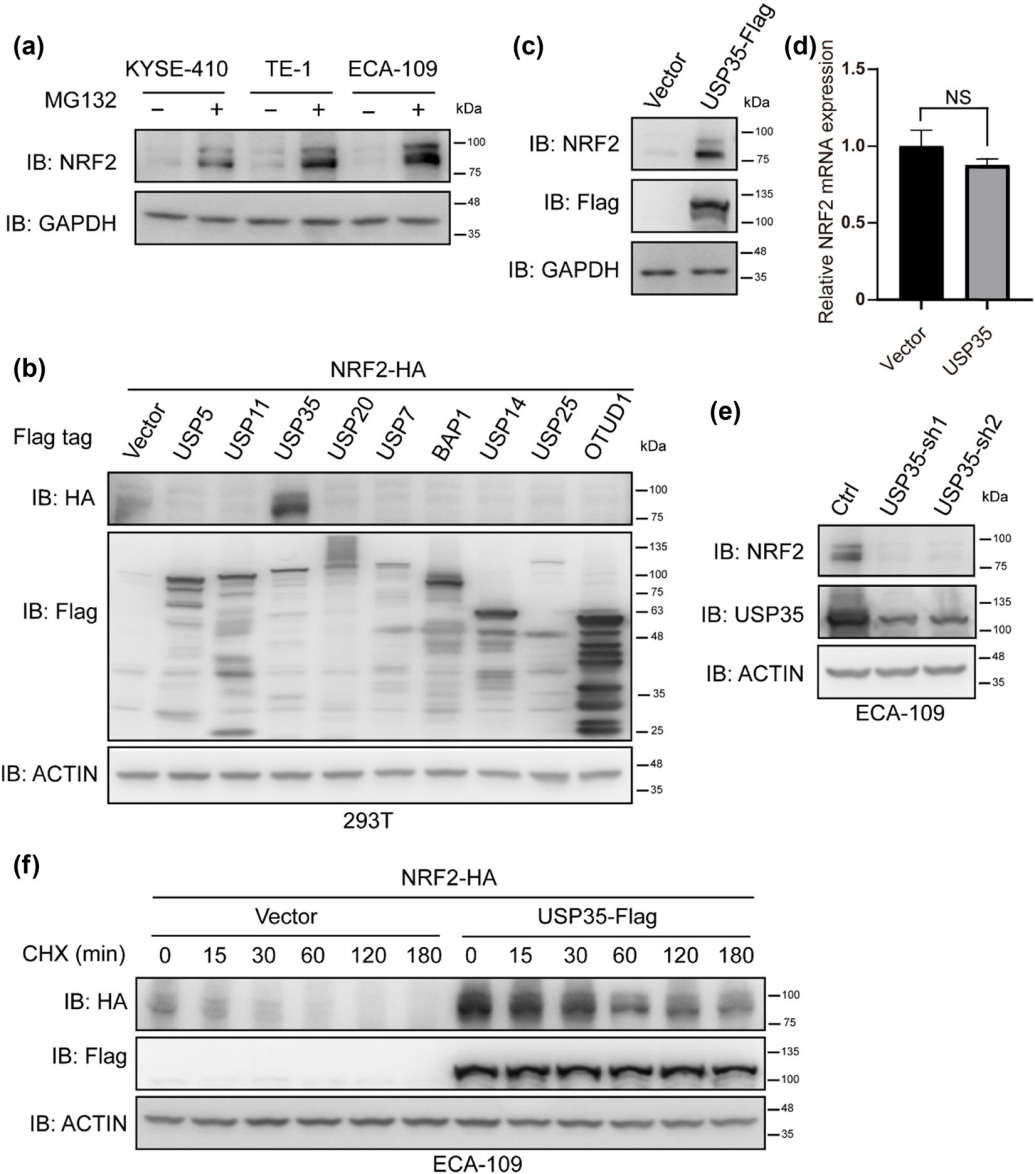
USP35 regulates the stability of NRF2 protein. (a) The proteasome inhibitor MG132 (10 μM) was applied to KYSE-410, TE-1, and ECA-109 cell lines for a duration of 6 h, followed by SDS-PAGE and western blot analysis using appropriate antibodies. (b) Plasmid expressing NRF2-HA was co-transfected with individual DUB expression plasmids into 293T cells. After 36 h, the cells were collected and analyzed using relevant antibodies. (c) Lentivirus expressing either empty or USP35-Flag was utilized to infect ECA-109 cells, which were subsequently harvested and analyzed with the appropriate antibodies. (d) NRF2 RNA levels were evaluated in ECA-109 cells infected with lentivirus carrying either empty or USP35-Flag constructs. (e) Lentivirus containing scramble or USP35-shRNAs was employed to infect ECA-109 cells, followed by cell harvesting and analysis using specific antibodies. (f) In TE-1 cells, co-transfection of a plasmid expressing NRF2-HA with either an empty vector or USP35-Flag expression plasmids was performed. Subsequently, the cells were treated with CHX (40 µg/mL) for a specified period, followed by cell harvesting and analysis using the designated antibodies after 24 h.

In order to investigate the regulation of NRF2 stability by DUBs, we conducted experiments to determine if NRF2 protein degradation occurs in an ubiquitination/proteasome-dependent manner. Our findings, illustrated in Figure 1a, demonstrate that treatment with the proteasome inhibitor MG-132 led to a significant increase in NRF2 protein levels in esophageal cancer cell lines. To identify specific DUBs involved in the regulation of NRF2 protein degradation, we co-transfected NRF2 with expression plasmids encoding individual DUBs in 293T cells and assessed their impact on NRF2 protein levels. Our results indicate that the expression of USP35, a DUB, significantly enhanced NRF2 protein levels (Figure 1b). Furthermore, the upregulation of USP35 was found to elevate endogenous NRF2 protein levels while having no impact on NRF2 RNA levels (Figure 1c and d). Conversely, downregulation of USP35 resulted in decreased NRF2 protein levels (Figure 1e). To validate the role of USP35 in modulating NRF2 protein stability, TE-1 cells were co-transfected with USP35 and NRF2 and treated with the eukaryotic protein synthesis inhibitor CHX. The results depicted in Figure 1f demonstrate that USP35 expression significantly enhances NRF2 protein stability.

### 3.2 Enzymatic activity of USP35 is significant in its regulation of NRF2

**Figure 2:**
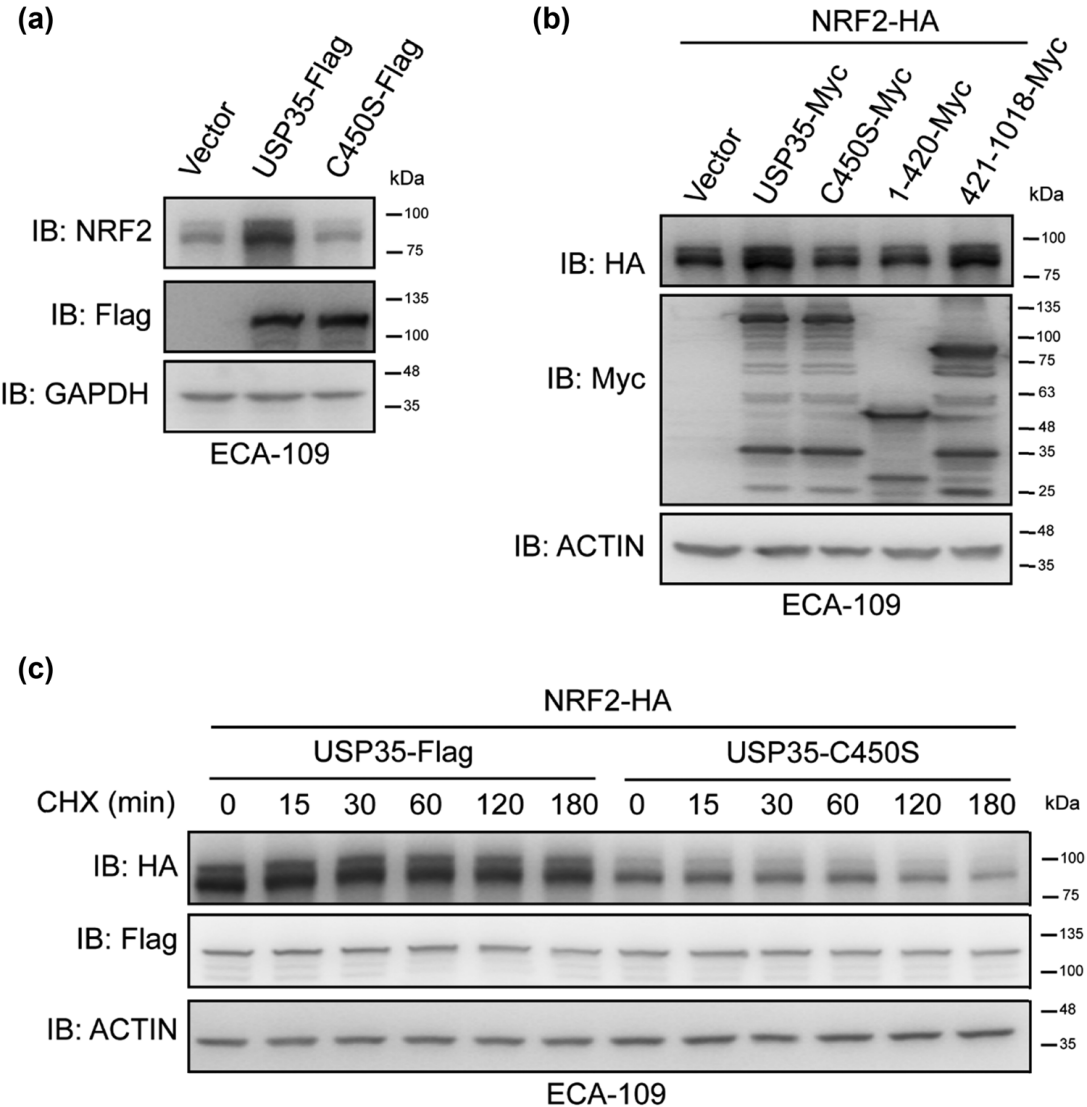
Enzymatic activity of USP35 is important in its regulation of NRF2. (a) Lentivirus expressing Vector, USP35-Flag, or USP35-C450S-Flag was utilized to transduce ECA-109 cells, followed by cell harvesting and analysis using specific antibodies. (b) Plasmid expressing NRF2-HA was co-transfected with plasmids expressing an empty vector, USP35, or its variants into ECA-109 cells. Subsequently, the cells were harvested after 36 h and subjected to analysis using the appropriate antibodies. (c) Plasmid encoding NRF2-HA was co-transfected with plasmids encoding an empty vector, USP35-Flag, or USP35-C450S-Flag into ECA-109 cells. Following a 24 h incubation period, the cells were exposed to CHX at a concentration of 40 µg/mL for designated durations. Subsequently, the cells were harvested and subjected to immunoblot analysis using the specified antibodies.

Subsequently, our investigation focused on the role of USP35 in regulating the protein stability of NRF2. Given the importance of a DUB’s enzymatic activity for its function, we conducted experiments to determine if USP35’s enzymatic activity impacts the stability of NRF2 protein. Specifically, we examined the effects of USP35-C450S, an enzymatically inactive mutant of USP35 characterized by a serine mutation at amino acid position 450. As shown in Figure 2a, only wild-type USP35, and not its enzymatically inactive mutant, was able to increase NRF2 protein levels. Subsequently, our investigation focused on determining the specific domain of USP35 responsible for regulating the stability of NRF2. Our findings indicate that only the USP35 construct containing its enzymatic domain (amino acids 421-1018), as opposed to the construct containing amino acids 1-420, exhibited a significant increase in NRF2-protein levels compared to full-length USP35 (Figure 2b). Furthermore, the stability of the NRF2 protein was enhanced by USP35, but not by its enzymatically inactive mutant (Figure 2c). Collectively, these results suggest that USP35 influences NRF2 protein stability through enzymatic mechanisms.

### 3.3 USP35 interacts with NRF2 and promotes NRF2 deubiquitination

**Figure 3:**
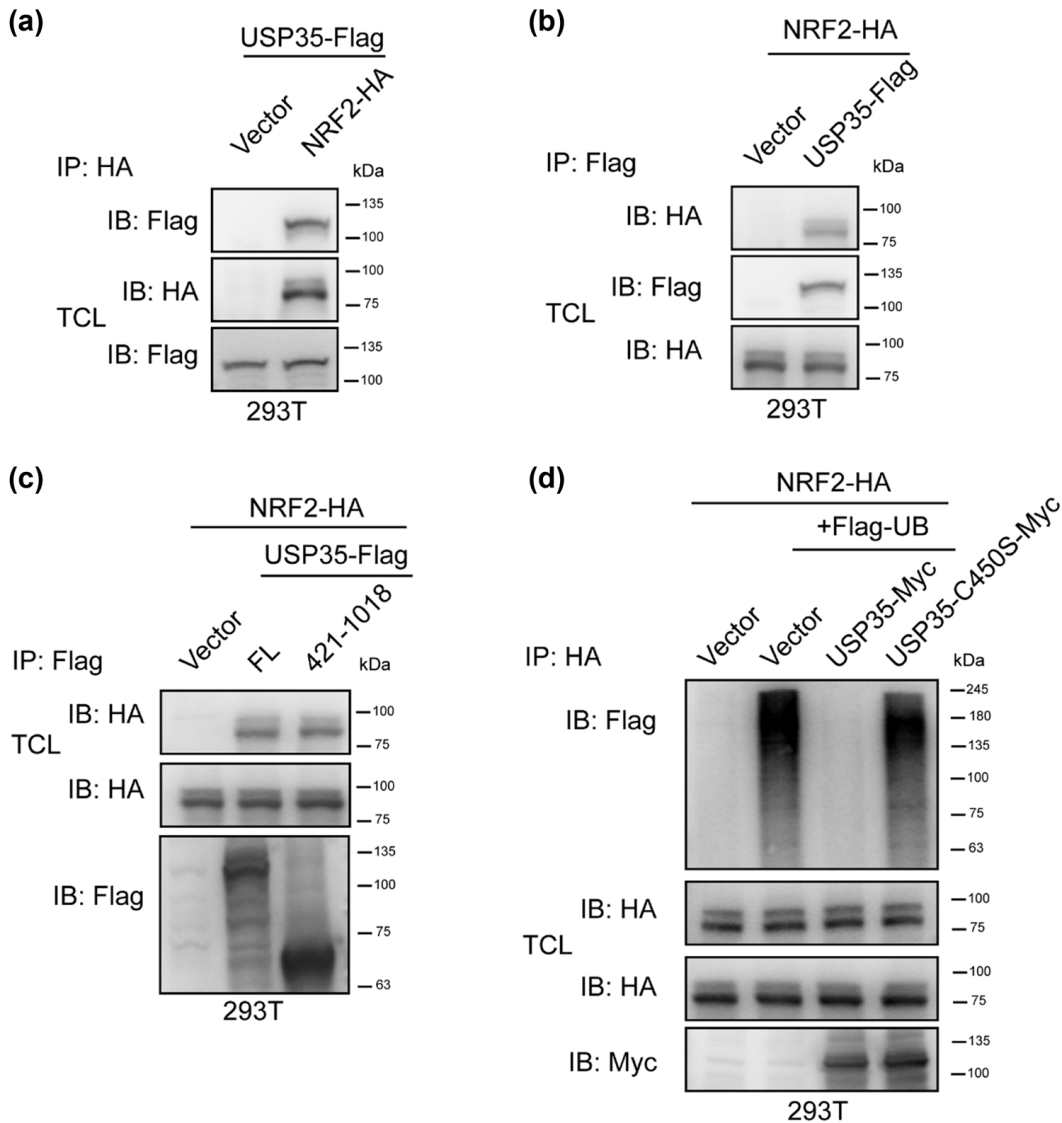
USP35 interacted with and promoted NRF2 deubiquitination. (a) An empty vector or NRF2-HA plasmid was co-transfected with a USP35-Flag plasmid into 293T cells. Prior to cell harvesting, the cells were treated with MG132 (10 µM) for a duration of 6 h. Subsequently, the cell lysates underwent immunoprecipitation utilizing the designated antibodies. Immunoblotting was carried out on both the total cell lysates and the immunoprecipitates using anti-HA or Flag antibodies. (b) Similarly to (a), with the exception that an empty vector or USP35-Flag plasmid was co-transfected with an NRF2-HA plasmid into 293T cells. (c) In a manner analogous to (a), the co-transfection of empty vector, USP35-Flag, or the enzymatic domain (aa 421-1018) plasmid with NRF2-HA plasmid was conducted in 293T cells. (d) Co-transfection of empty vector, USP35-MYC plasmid, or USP35 enzymatic mutant C450S-MYC plasmid with NRF2-HA and Flag-ubiquitin (Flag-Ub) plasmids was carried out in 293T cells. Subsequently, the cells were treated with MG132 (10 µM) for 6 h prior to harvesting. Immunoprecipitation was performed on the cell lysates using anti-HA antibody, followed by immunoblotting using Flag (Flag-UB), HA (NRF2-HA), and Myc (USP35-Myc) antibodies on both the lysates.

Subsequently, an investigation was conducted to determine the potential interaction between USP35 and NRF2 in order to elucidate the mechanism by which USP35 regulates the protein stability of NRF2. The results presented in Figure 3a and b demonstrate a mutual interaction between USP35 and NRF2. Further analysis was carried out to ascertain the specific protein domains through which USP35 and NRF2 interact. As illustrated in Figure 3c, it was observed that NRF2 is capable of interacting with enzymatic domains of USP35 (421-1018). As numerous DUBs are known to modulate protein stability by cleaving ubiquitin chains from substrates, our study investigated the potential impact of USP35 on the ubiquitination of NRF2. Our findings, as depicted in Figure 3d, demonstrate that USP35 specifically targets the ubiquitin chain of NRF2, but not that of its inactive mutant. In conclusion, our results suggest that USP35 interacts with NRF2 and deubiquitinates it, thereby regulating its protein stability.

### 3.4 USP35 causes chemotherapy resistance by upregulating NRF2 in ECA-109 cells

**Figure 4:**
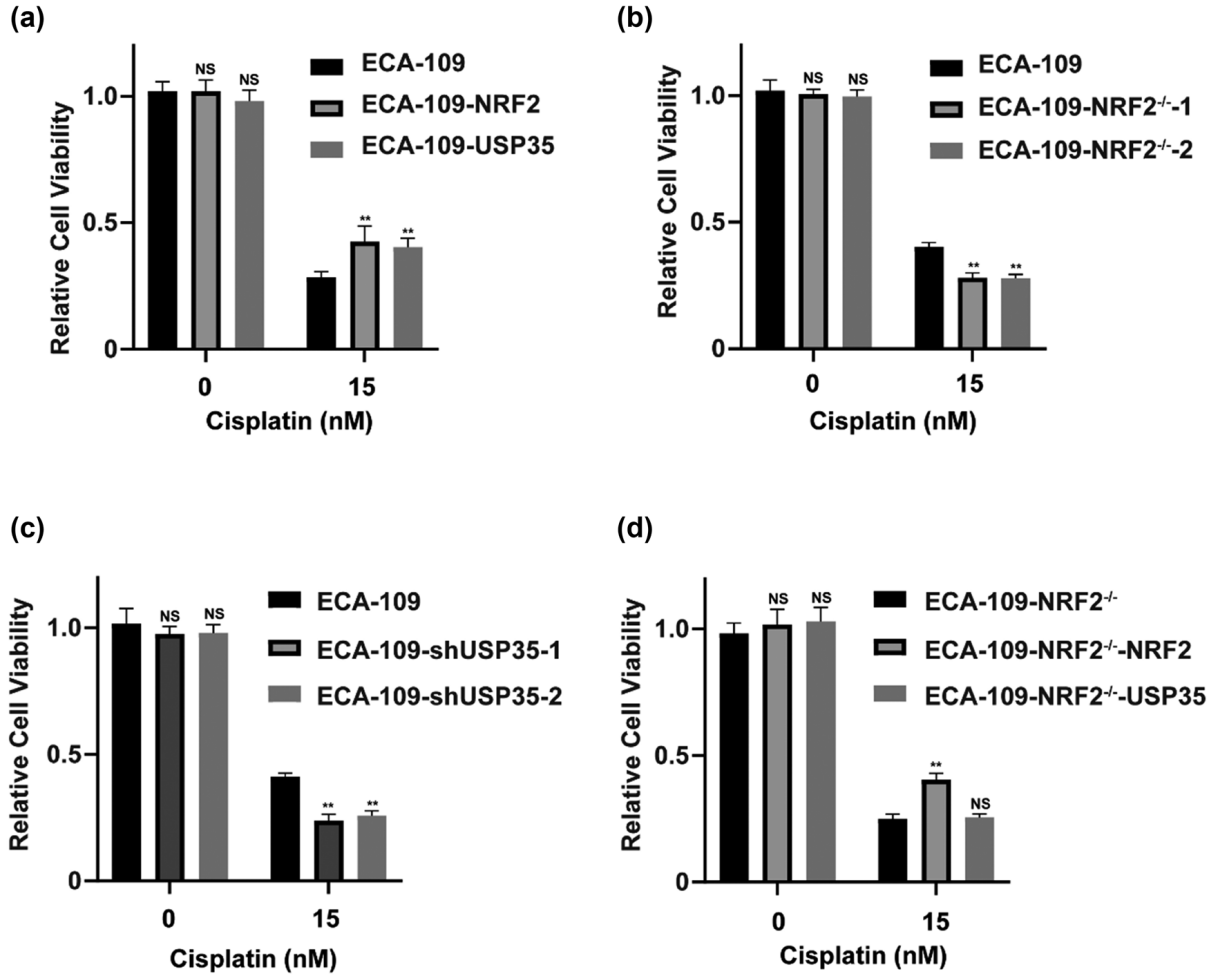
USP35 causes chemotherapy resistance by upregulating NRF2 in ECA-109 cells. (a)–(d) USP35 has been identified as a contributor to Cisplatin resistance through the upregulation of NRF2. To investigate this mechanism, we generated stable cell lines expressing ECA-109-vector, ECA-109-NRF2, and ECA-109-USP35, as well as KO cell lines ECA-109-NRF2−/−-1 and ECA-109-NRF2−/−-2, and knockdown cell lines ECA-109-shNC, ECA-109-shUSP35-1, and ECA-109-shUSP35-2. The ECA-109-NRF2−/− cell line was transduced with lentivirus containing NRF2 or USP35 to establish stably rescued cell lines designated as ECA-109-NRF2−/−-NRF2 and ECA-109-NRF2−/−-USP35. These cell lines were then subjected to treatment with varying doses of Cisplatin for 48 h in a 96-well plate, followed by measurement of cell viability.

A key emphasis of this research is the examination of drug resistance within the realm of cancer therapeutics. Previous research has demonstrated that the expression of the NRF2 protein is linked to the development of resistance to chemotherapy in cancer cells. The data presented indicate that USP35 acts as a novel deubiquitinase of NRF2. Notably, the upregulation of USP35 in various cancers implies a potential relationship between USP35 and NRF2 in the context of resistance to chemotherapeutic agents. To investigate this hypothesis, three stable ECA-109 cell lines were utilized, each expressing either an empty vector, NRF2, or USP35. Through the utilization of sell viability assays, our study examined the impact of Cisplatin, a chemotherapy agent, on esophageal cancer cells. Our findings indicate that the upregulation of NRF2 or USP35 diminishes the cytotoxic effects of Cisplatin on esophageal cancer cells (Figure 4a). Furthermore, a notable enhancement in susceptibility to Cisplatin treatment was observed in ECA-109 cells with NRF2 KOs and USP35 knockdowns (Figure 4b and c). The sensitization effects induced by NRF2 KO were reversible upon NRF2 re-expression, but not by USP35 (Figure 4d), suggesting that USP35 contributes to resistance against Cisplatin.

## 4 Discussion

A significant portion of the ARE signaling pathway is mediated by NRF2 and its downstream effector genes, leading to the activation of numerous transcription factors. The NRF2-ARE signaling pathways are closely linked to various pathophysiological processes, such as metabolism and oxidative stress. Our study identifies USP35 as a prominent candidate in the screening process and highlights its extensive investigation into the molecular mechanisms governing its regulatory role in the NRF2-ARE signaling pathway in esophageal cancer cells. In our research, we found that USP35 interacts with NRF2 and aids in its deubiquitination, leading to the inhibition of its proteasomal degradation.

In numerous cellular processes, ubiquitination-proteasome degradation and the DUB system play vital roles in regulating transcription, DNA repair, signal transduction, cell cycle control, protein degradation, and cancer development. Nevertheless, additional research is needed to fully understand the relationship between the deubiquitinase USP35 and cancer chemotherapy. Given the association between NRF2 and chemoresistance, our study aimed to illustrate the role of USP35 in regulating chemoresistance through deubiquitination and stabilization of NRF2. Our findings indicate that depletion of USP35 significantly enhances Cisplatin-induced cell death in esophageal cancer cell lines. Consequently, our research will pave the way for further exploration into the deubiquitination mechanisms and regulatory pathways of NRF2. In conclusion, our results suggest that USP35 enhances chemotherapy resistance by deubiquitinating and stabilizing NRF2, offering a promising avenue for addressing chemoresistance in esophageal cancer through NRF2 activation.

Numerous antioxidant pathways, such as the NRF2-ARE antioxidant response pathway, are crucial in cancer therapy and chemoprevention. Thus, a stringent regulatory framework is necessary to impede the onset and advancement of cancer. Research indicates that cancer cells exhibiting elevated NRF2 levels demonstrate reduced sensitivity to chemotherapy [25]. The NRF2 protein is a transcription factor, and previous studies have shown that NRF2 is deubiquitinated by a variety of DUBs, such as DUB3, USP11, and USP35 [26–28]. Diverse DUBs play a key role in various tumors, which implies that different DUBs may be active in different tumors to regulate the stability of the NRF2 protein. For instance, the heterotopic expression of DUB3 has been shown to result in NRF2-dependent chemotherapy resistance in colon cancer cell lines [26]. Depletion of USP11 has been found to inhibit cell proliferation and induce ROS-mediated stress-induced iron cell death [27]. Silencing of USP35 leads to a reduction in NRF2 levels, thereby increasing the sensitivity of renal clear cell cancer cells to iron death induction [28]. Our research indicates that knockdown of USP35 results in a significant elevation of NRF2 levels and enhances the sensitivity of esophageal cancer cells to chemotherapy. Previous research has indicated that USP11 plays a role in stabilizing the NRF2 protein. However, our investigation into the regulation of NRF2 protein stability did not find evidence of USP11’s involvement in this process. This discrepancy may be attributed to the use of foreign expression vectors or screening methods. Both our study and the study by Wang et al. identified USP35 as a deubiquitination enzyme present in various tumors, suggesting that this enzyme may stabilize the same substrate protein in different tumor types to facilitate its function. Our study primarily investigates chemotherapy resistance as a significant barrier to effective treatment of esophageal cancer, with the aim of identifying key factors contributing to drug resistance. In our functional analysis, cells that overexpressed NRF2 and USP35 exhibited a survival rate approximately 10% lower than cells that did not overexpress these proteins. This is attributed to the presence of additional DUBs and drug-resistant molecules within esophageal cancer cell lines, which are implicated in the resistance to chemotherapy drugs. This observation further indicates the existence of untapped targets for addressing tumor resistance.
